# Systematic Review of Self-Management Assessment Tools for Children With Inflammatory Bowel Disease

**DOI:** 10.1097/PG9.0000000000000075

**Published:** 2021-05-27

**Authors:** Angharad Vernon-Roberts, Emma Rouse, Richard B. Gearry, Andrew S. Day

**Affiliations:** From the * Department of Paediatrics, Otago University, Christchurch, New Zealand; † Department of Medicine, Otago University, Christchurch, New Zealand.

**Keywords:** self-management skills, ulcerative colitis, Crohn’s disease, validity, reliability, readability, health literacy, self-efficacy, transition

## Abstract

The relapsing pattern of illness and diverse management strategies for children with inflammatory bowel disease (IBD) may inhibit their opportunities to develop self-management skills. Interventions focused on the development of these skills require population-specific outcome measures. We provide a comprehensive overview of self-management skills assessment tools that are available for children with IBD that target processes and behaviors outlined as beneficial for the development of these skills. Seven health literature databases were searched using terms related to self-management, transition, IBD, Crohn’s disease, ulcerative colitis, and children. Identified studies underwent a process of title and then full text review. Included studies underwent data extraction using a priori quality assessment criteria related to self-management skills, health literacy, suitability for the target population, validity and reliability testing. The searches identified 523 papers, 123 of which underwent full-text review and 10 included in the final analysis. The assessment tools identified showed great diversity in their development approach, content, methodological rigor, and health literacy considerations. The most frequently included self-management skills were related to adherence, communication, attending clinic, and disease/treatment knowledge. None of the tools satisfied the criteria of selection for use in the target population. While many chronic diseases of childhood follow a predictable disease course with established treatment pathways, IBD presents a unique set of challenges. The development of self-management skills is vital for successful disease management, but these skills should be assessed by a tool appropriate for the population and their diverse needs.

## Abstract 2

Supplemental Digital Content is available in the text.

**What Is Known**

- The development of self-management skills begins before adolescence.
- The unique, individual nature of inflammatory bowel disease necessitates diverse and inclusive self-management skills.
- Health literacy is an important consideration for tools aimed at children.

**What Is New**

- Generic assessment tools may not be adequate to measure the multiplicity of inflammatory bowel disease self-management skills.
- Assessment tools should be based on an empirical framework to address the processes and behaviors integral to the development of self-management skills.

## INTRODUCTION

Inflammatory bowel disease (IBD) is a chronic condition encompassing 2 clinical subtypes; Crohn’s disease and ulcerative colitis, with an increasing global incidence among the pediatric population (1). IBD is characterized by a relapsing and remitting pattern of illness that is unique and unpredictable for each patient. Children with IBD often require diverse management strategies and experience adverse outcomes of their condition that influence their developmental trajectories across a multitude of social, physical, and psychological domains (2). Disease management strategies that address these factors concurrently are considered beneficial, and interventions focusing on self-management (SM) demonstrate positive effects across a number of disease outcomes for adults with IBD. These include improved quality of life, improved adherence, symptom reduction, reduced disease exacerbations, and lower health care utilization (HCU) (3–7).

SM for children with IBD is still a nascent field, but is considered to be an ongoing process toward achieving control of their condition through performing a range of skills and activities (8). When delivering interventions targeted at SM efficacy should be assessed using population appropriate outcome measures. However, the complex and relapsing nature of pediatric IBD may exclude the use of generic tools that fail to address the unique characteristics of the disease, its treatment modalities, and the range of skills required by children to manage their condition.

We conducted a systematic review to identify reports containing tools for the assessment of self- management skills for children with IBD aged 10 and over, the approximate age it is considered SM skills begin to develop. Our secondary aim was to determine which identified tool was most appropriate for evaluating SM skills in this population.

## METHODOLOGY

The systematic review protocol, search strategy, and implementation were performed using the Preferred Reporting Items for Systematic Reviews and Meta-Analyses guidelines (9).

### Search Strategies

The following databases were searched: Medline, Embase, Cumulative Index to Nursing and Allied Health Literature, PsychInfo, Cochrane database, Scopus, and the Joanna Briggs Institute. The individual search strategies are included (Supplemental Digital Content Appendix 1, http://links.lww.com/PG9/A29), but the main terms included were related to SM, transition, IBD, Crohn’s disease, ulcerative colitis, and children. Additional search limits were applied for the year 1998 onward as while the theory of pediatric SM began in the 1960s (10), it was considered that medical care and expectations for SM have changed most significantly in the last 20 years, alongside the increased incidence of pediatric IBD.

### Paper Selection and Data Extraction

All identified papers were synthesized into a database, the duplicates removed, and the remaining titles examined by 2 reviewers (A.V.-R. and E.R.) to identify those relevant for a full text review. Disputes were resolved by discussion between 3 reviewers (A.V.-R., E.R., and A.S.D.). All relevant articles were read in full text by 2 reviewers (A.V.-R. and E.R.), and those not considered were categorized with a reason for exclusion. For the remaining articles, the details of the paper and the included SM evaluation tool were extracted to address the quality assessment criteria.

### Quality Assessment Criteria

Criteria for selection of a SM skills assessment tool for use with children with IBD focused on a number of factors.

### SM Skills

Multicomponent interventions for children with IBD should contain all domains known to collectively contribute to the development of SM skills. These have been categorized in the literature as knowledge, self-regulation, adherence, cognitive attributes, and communication (11–14). Further, a number of individual processes and behaviors have also been identified as beneficial to the development of SM skills within these domains, as set out in the Pediatric Self-Management Framework developed by Modi et al and supported by further literature (11–14). These include taking drugs/treatment, knowledge, attending clinic, communication, refill prescriptions, behavioral compliance, seeking information, symptom management, self-efficacy, determining health care needs, self-care, lifestyle modifications, HCU. The identified assessment tools were therefore required to address these components.

The critique of the identified articles included an assessment of whether the tool they contained was classified as relating to SM, transition, or self-efficacy. All may contain items for assessing practical SM skills; however, transition tools may have items that concentrate on topics that may be too mature for children aged 10 years. In addition, there is a distinct cross-over between self-efficacy and SM, with the former referring to an individual’s perception and belief of their ability to engage in IBD SM behaviors and may, therefore, be measured in a different way to practical skills (15, 16).

### Health Literacy

The target population were children over the age of 10 years, and therefore attention was given to factors appropriate for populations with low health literacy: brevity, simplicity, and readability (17, 18). This was to ensure that the respondent burden of length, complexity, and comprehension were deliberated.

#### Length

The ideal number of items for an assessment tool such as this has not been established, but it has been suggested that the response burden imposed by increasing survey lengths may result in lower response rates, reduced completion, and reduced data quality (17).

#### Simplicity

Assessment of simplicity for tools aimed at a target audience of children, such as the question format, and the way in which they can be answered can be accomplished in several ways. Compound items, whereby more than 1 query is combined into a single question, may cause confusion when the format of answering includes a Likert scale that may warrant a different answer for each query. The type of scoring scale used to elicit an answer and the number of response options are also relevant factors, as children prefer Likert scales to Visual analog scales (19), prefer text Likert scales to numbered Likert scales (20), and prefer 3 Likert options to 5 (21).

#### Readability

The importance of readability and comprehension for material and assessment tools aimed at children has been discussed in the literature (22–24), yet readability levels are infrequently reported. Reading comprehension assessments determine the complexity level a text should have for a reader to understand the written material, and the resulting score equates to age and formal years of schooling that a reader requires to understand the text (18). The recommendations are that for low health literacy groups such as children, health care literature should be written at or below a grade 5 (age 10 years) reading level (18).

### Appropriate for Children With IBD

To ensure relevance to the target population, the tool was required to have been tested among children with IBD to ensure that the unique characteristics of the disease did not preclude it from use.

### Generalizability

The tool was required to be generalizable to all English-speaking countries; therefore, items relating to specific health care systems were reviewed for exclusivity. Tools containing items applicable to older children (eg, pregnancy, smoking, and alcohol) would be reviewed for their appropriateness for children aged 10 years and over.

### Validity and Reliability

To ensure methodological rigor the identified tool must have been validated, and preferably undergone reliability testing.

## RESULTS

### Article Selection Process

**FIGURE 1.**
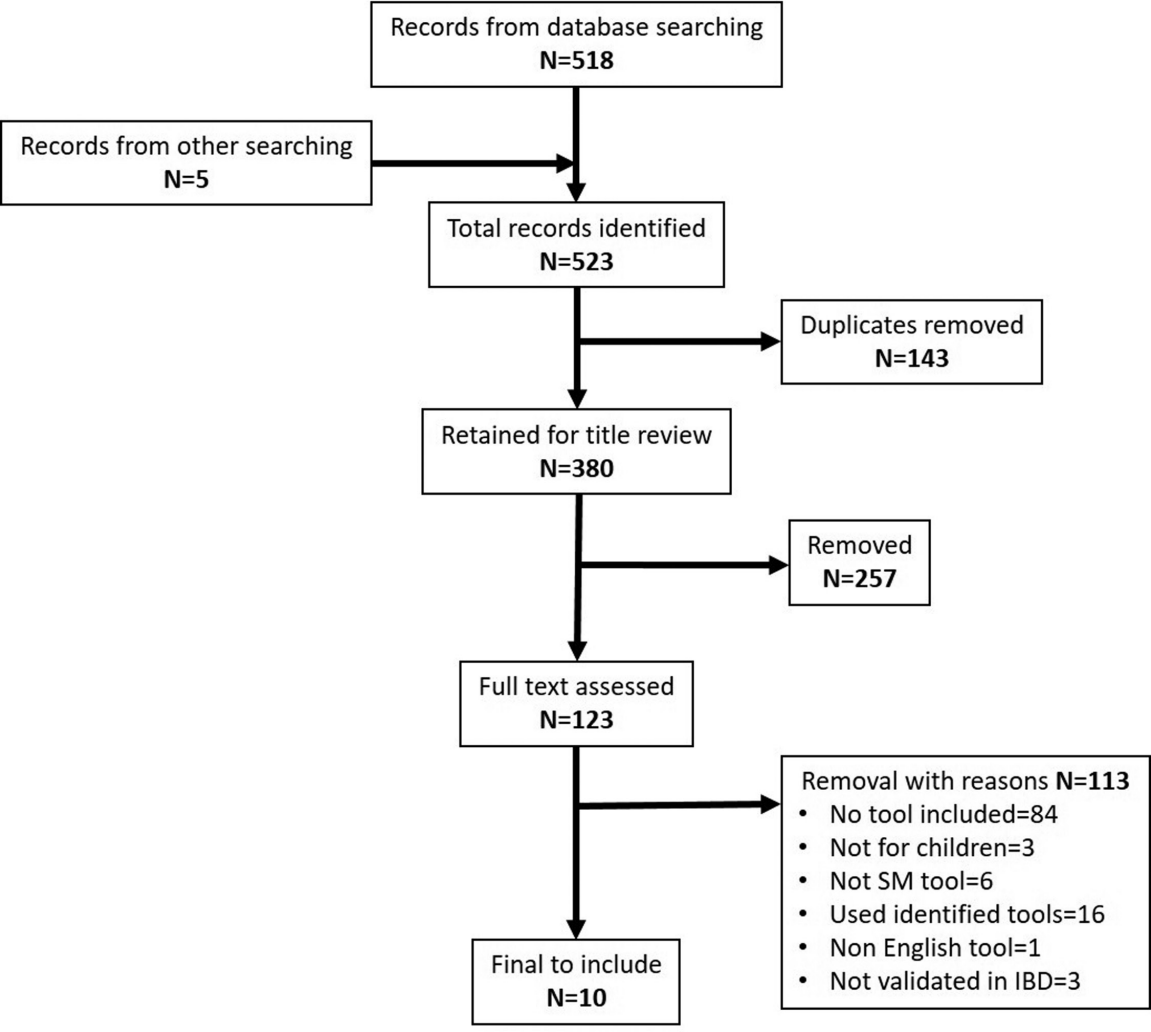
PRISMA flow chart of the article selection process. IBD = inflammatory bowel disease; N = number; PRISMA = Preferred Reporting Items for Systematic Reviews and Meta-Analyses; SM = self-management.

Five hundred and twenty-three publications were identified, and 10 met the inclusion criteria of containing a SM skills assessment tool for children with IBD (Fig. 1).

### Study Characteristics

The identified studies presented a variety of approaches to measuring SM across a wide age range of participants. Development methods were highly variable, as was the methodological rigor used for testing reliability and validity. When summarized, it was possible to examine the basic attributes of each tool before an in-depth evaluation (Table 1). In the simplest form, the assessment tools presented by Hait et al (25) and the North American Society of Gastroenterology, Hepatology and Nutrition (NASPGHAN) (30) were transition checklists that had been developed from literature review, expert opinion, and anecdotal evidence. These tools were both divided into sets of transition skills that could be expected to be attainable by specified ages, and both included a set of expectations for both the patient and healthcare team.

**TABLE 1.** SM assessment tool characteristics

First author	Year	Country of origin	Development process	Topic(s)	Participant ages, y	Participant numbers	Format	Likert options	Likert format	Items	S or C items	Validity tested	Reliability tested	Readability tested
Hait et al (25)	2006	US	Author written	IBD, T	11–23	N/A	Checklist	-	-	17	C	No	No	No
Fishman et al (26)	2010	US	Author written	IBD, SM, T	16–18	40	Survey Likert	5	Help	19	S	No	No	No
Zijlstra et al (27)	2013	NDL	Literature synthesis	IBD, SE	14–18	50	Likert VAS	4	Mixture	74	S	No	Yes	No
Whitfield et al (28)	2015	US	Other author	IBD, SM	10–21	67	Likert	3	Help	23	C	No	No	No
Izaguirre et al (29)	2014	US	Qual PRO	IBD, SE	10–25	95	Likert	5	Agreement	13	C	Yes	Yes	Yes
NASPGHAN (30)	2010	US CAN	Author written	IBD, T	12–17	-	Checklist	-	-	27	C	No	No	No
Klassen et al (31)	2014	CAN	Qual PRO	SM, T	12–18	337 (71 IBD)	Likert	3	Frequency	14	S	Yes	Yes	Yes
Ferris et al (32)	2015	US	Qual Quant	SM, T	12–25	194 (57 IBD)	Likert	5	Frequency	18	S	Yes	Yes	Yes
Ferris et al (33)	2012	US	Qual Quant	SM, T	12–20	92 (46 IBD)	Survey Likert	3	Mixture	33	C	Yes	Yes	No
Williams et al (34)	2010	CAN	Literature Synthesis	SM, T	11–18	49 (5 GI)	Likert	5	Agreement	21	C	Yes	Yes	Yes

C = compound; CAN = Canada; GI = gastrointestinal; IBD = inflammatory bowel disease; NDL = Netherlands; PRO = patient-reported outcome measure; Qual = qualitative research; Quant = quantitative research; S = simple; SM = self-management; SE = self-efficacy; T = transition; US = United States; VAS = visual analog scale.

Fishman et al (26) and Whitfield et al (28) presented transition and SM assessment tools for children to report whether SM tasks could be performed by the participants on their own, or with varying levels of help. The tool presented in the Whitfield et al (28) article was devised by the ImproveCareNow network in the United States (35) and is included in their SM manual (36).

Two studies by Zijlstra et al (27) and Izaguirre et al (29) assessed self-efficacy. Zijlstra et al’s (27) “IBD-yourself” tool also assessed independence and disease burden and was developed by the authors using a synthesis of the available literature. Izaguirre et al’s (29) “IBD Self-efficacy Scale for Adolescents and Young Adults (IBDSES-A)” was developed using qualitative interviews carried out with the target population to produce a patient-reported outcome measure (15).

The final 4 studies contained generic transition and SM assessment tools that consisted of the Successful Transition to Adulthood with Therapeutics [Rx] (STARx) Questionnaire (32), the University of North Carolina (UNC) TRxANSITION Scale (33), a tool developed for the Alberta Children‘s Hospital and tested by Williams et al (34), and the TRANSITION-Q by Klassen et al (31). All 4 tools were developed to assess the child’s ability to perform set tasks. The TRANSITION-Q was developed as a patient-reported outcome; the 2 Ferris tools, UNC TRxANSITION scale and STARx, were developed using a mixture of qualitative and quantitative methods, and the Alberta Children‘s Hospital tool was developed using a synthesis of other available tools.

### SM Skills

The degree to which each tool identified individual processes and behaviors from the pediatric SM skills framework (12) were assessed (Table 2). The most frequently represented of the 11 SM elements was taking drugs/treatment, and the least frequently represented were lifestyle modifications and HCU. The tool presented by Whitfield et al (28) contained the highest number of SM elements, and the lowest was the IBDSES-A by Izaguirre et al (29).

**TABLE 2.** Frequency of the self-management processes contained in the identified tools

Self-management skill	Ferris et al (33)	Ferris et al (32)	Zijlstra et al (27)	Hait et al (25)	Klassen et al (31)	NASPGHAN (30)	Izaguirre et al (29)	Fishman et al (26)	Whitfield et al (28)	Williams et al (34)
Taking drugs/treatment	✓	✓	✓	✓	✓	✓	✓	✓	✓	✓
Personal disease/treatment knowledge	✓	✓	✓	✓	✓	✓		✓	✓	✓
Attending clinic	✓	✓	✓	✓	✓	✓		✓	✓	✓
Communication with the medical team	✓	✓	✓	✓	✓	✓		✓	✓	✓
Refill prescriptions	✓		✓	✓	✓	✓		✓	✓	✓
Behavioral compliance		✓	✓	✓		✓	✓	✓	✓	✓
Seeking disease/treatment information		✓		✓	✓	✓	✓	✓		
Self-management of symptoms			✓				✓		✓	
Self-efficacy		✓	✓				✓			✓
Determining health care needs					✓				✓	✓
Self-care	✓					✓				
Lifestyle modifications									✓	
Health care utilization									✓	
Totals	6	7	8	7	7	8	5	7	10	8

✓ indicates a process was included; blank space indicates it was excluded.

### Health Literacy

#### Length

The studies by Zijlstra et al (27) and Ferris et al (33) had the greatest number of items (74 and 33, respectively) while also missing up to 4 of the required behaviors and processes from the Modi et al (12) framework (Table 2). The shortest assessment tools were by Klassen et al (31) and Izaguirre et al (29) (14 and 13 items, respectively), which were a more appealing survey length, but were missing up to 7 of the SM elements. The tools of Whitfield et al (28) and NASPGHAN (30) (27 and 23 items, respectively) both included the highest numbers of SM behaviors and processes.

#### Simplicity

Five of the tools included several compound items. One assessment tool used a visual analog scale, in combination with Likert scales (27), and 6 other tools included Likert scales. The Likert scales presented were asking respondents to rate different concepts: agreement with a statement about SM tasks (29), the frequency of performing SM tasks (31, 32), needing help to do SM tasks (26, 28), and a mixture of Likert response options (27, 33). Three of the tools included 5 Likert response options (26, 29, 32), 1 included 4 (27), and 3 had 3 options (28, 31, 33).

#### Readability

Among the tools in this review, readability was referenced in just 3 studies. Izaguirre et al (29) mentioned that the readability/clarity of IBDSES-A had been assessed by participants during the development process, but no data were included in the results. Ferris et al (32) reported an overall Flesch-Kincaid readability Grade level of 4.4 for their *STARx* tool, indicating that this tool had an acceptable level of readability for a 9-10 year old (37). Klassen et al (31) reported a Flesch-Kincaid readability Grade level for every item in the *TRANSITION-Q,* at each stage of development and following revisions. The overall reported grade level for the whole tool was also 4.4 - an acceptable level of readability for the target population of this review.

### Appropriate for Children With IBD

Six of the ten tools were developed specifically for the pediatric IBD population (25–30), 3 more included a cohort of children with IBD in their testing schedule (31–33), and one stated they included children with a ‘gastrointestinal’ condition (34). This latter study by Williams et al (34) was included in this review as the chronic gastrointestinal conditions experienced during childhood and adolescence that require a structured SM or transition strategy are mainly limited to IBD. Other possible conditions would not have the same SM requirements (38), and it was considered likely the study cohort included children with IBD. In addition, the centre where the tool was developed have a structured transition programme for children with IBD but not for other chronic gastrointestinal conditions (39). The authors of the paper were contacted for clarification of this point, but no response was received.

### Generalizability

Four tools contained items regarding health insurance (25, 30, 33, 34) and therefore have limited generalizability for use in other countries with different health care systems. Six of the tools contained items relating to smoking, drugs, pregnancy, sex, or alcohol and would, therefore, not be appropriate for the target population of children aged 10 years and over (25, 27, 28, 30, 33, 34).

### Validity and Reliability

No validation or reliability testing was performed on the checklists by Hait (25) or NASPGHAN (30), or on the Fishman (26) and Whitfield (28) tools.

The self-efficacy scale *IBDSES-A* (29) measured reliability, as well as concurrent validity against established measures of self-esteem, depression, anxiety, and quality of life. *IBD- yourself* (27) was tested for reliability but not validity, however, parents and clinicians completed a modified assessment for comparison.

Reliability and concurrent, predictive and discriminant validity testing was performed on the *STARx* (32, 40). Reliability and inter-rater reliability was tested for the *UNC TRxANSITION scale* (33), and construct and content validity was inferred based on the development process. The Alberta Children‘s Hospital tool (34) had reliability assessed, and concurrent validity against a scale of functional independence. Construct validity for the *TRANSITION-Q* (31) was established using hypothesized score patterns and Rasch-based score testing, and reliability also assessed.

## DISCUSSION

The studies identified in this systematic review provided a small number of assessment tools that may be used to determine SM skills in children with IBD. Using pre- determined, empirically based criteria for the selection of an appropriate tool for use with the target population enabled an objective assessment for each one. The diversity in strengths and limitations precluded any from selection based on the following discussion.

The processes and behaviors that were poorly represented in the tools (lifestyle modifications, HCU, and self-care) warrant review to consider their importance for inclusion. Lifestyle modification opportunities in the pediatric IBD population are considerably different to adults and while the deleterious effects of smoking, alcohol and recreational drug use have been well studied (41–43), these factors are more appropriate for adolescents, not younger children. For younger children the main modifiable influences are stress (44, 45) and trigger foods (46, 47), both of which may cause exacerbation of symptoms and may be monitored through SM processes such as self-regulation.

The issue of HCU is highly relevant to the promotion of SM for IBD patients as medical costs per patient lifetime are suggested to be higher than those for diseases such as cancer and heart disease (48). Effective SM has huge cost-saving potential by reducing morbidity related to non-adherence or problematic SM (12). The poor representation of HCU in the pediatric tools may be attributed to the fact that HCU is not considered a ‘skill’ that children should master, and may also be problematic to measure as it relates to a number of skills, metrics, and adherence behaviors.

The opportunities for children with IBD to carry out self-care tasks are limited unless an individual need such as stoma care is required. This contrasts with, for example, children with cystic fibrosis who may learn to perform their own physiotherapy regimen, or children with type 1 diabetes who begin to monitor their own blood glucose levels. It was, therefore, considered that the selected tool was *not* required to contain items pertaining to these 3 elements.

Seven of the tools may have been appropriate for the target population following the exclusion of specific items regarding transition, smoking, drugs, pregnancy, sex, and alcohol. In addition, a number of tools contained items regarding health insurance and therefore have limited generalizability for use in other countries with different health care systems.

However, changing validated tools for one’s own purpose by removing items will render the tool, and any results produced, invalid. These 7 tools were, therefore, excluded from the selection (25, 27, 28, 30, 32–34). Of the 4 remaining tools, one had no validity testing and was excluded from the selection (26).

The 2 remaining tools both contained the smallest number of items and were lacking the highest numbers of required SM processes and behaviors. The self-efficacy tool *IBDSES-A* (29) had methodological rigor and good readability but contained compound items to be answered by a single Likert scale. In addition, the tool lacked some of the aspects considered crucial to SM in children with IBD: knowledge, attending appointments, and communication. It was therefore excluded from selection. The *TRANSITION-Q* tool by Klassen et al (31) also had methodological rigor, but the tool was missing SM of symptoms and components relating to adherence and behavior compliance and was excluded from selection. Following these elimination criteria, no study remained that could be considered wholly appropriate for use with the target population of this review.

### Limitations

In developing search strategies that included tools for assessing transition readiness there was a risk that they may include items relevant to older children or adolescents. The tools containing these items were not exclusively categorized as transition specific, despite the fact that SM is customarily aimed at increasing responsibility for disease management in adolescents, not younger children.

In using the SM framework by Modi et al (12) to define the selection criteria it could be considered that this limited the scope of the analysis. However, a content synthesis of other pediatric SM frameworks was performed prior to the review that showed the Modi et al (12) framework to be a highly inclusive model that was only minimally augmented by the additional papers. Only one of the papers identified in this review referenced a theoretical framework, with the Ferris et al (33) UNC TRxANSITION scale being based on the Self Determination Theory. It was therefore considered important to have specific criteria against which the tools could be evaluated, and the processes and behaviors outlined in the Modi et al framework (12) were utilized for this purpose.

### Strengths

The identification of assessment tools from a variety of fields and focal points indicates that the search strategies were adequate and performed well. The evident cross-over between SM, transition, and self-efficacy resulted in a diverse selection for comparison with a number of different concepts being studied, each with their own SM criteria.

## CONCLUSION

While other chronic conditions of childhood may have a relatively predictable disease course, easily anticipated symptomatology, and well-established treatment pathways, IBD is unique and unpredictable for each individual. These factors can affect the extent that SM skills are required, or limit the opportunities for development, and it was important that any tool chosen from this review could adequately gauge the diversity of IBD management. No assessment tool satisfied all the criteria for inclusion as a suitable outcome measure for the target population. Following on from this systematic review, the assessment tools identified underwent a process of content synthesis to develop a novel, evidence-based, SM skills assessment tool specific to the population of children with IBD. This tool is called IBD-STAR and has undergone a process of validation (2) and an assessment of generalizability (study ongoing).

## ACKNOWLEDGMENTS

All named authors made substantial contributions to the conception or design of the work; or the acquisition, analysis, or interpretation of data for the work. All named authors revised the work critically for important intellectual content and gave approval for the final draft to be published. All named authors agree to be accountable for all aspects of the work in ensuring that questions related to the accuracy or integrity of any part of the work are appropriately investigated and resolved.
